# 16S and 23S rRNA Gene Mutation Independent Multidrug Resistance of Non-Tuberculous Mycobacteria Isolated from South Korean Soil

**DOI:** 10.3390/microorganisms8081114

**Published:** 2020-07-24

**Authors:** Hyun-Eui Park, Suji Kim, Soojin Shim, Hong-Tae Park, Woo Bin Park, Young Bin Im, Han Sang Yoo

**Affiliations:** 1 Department of Infectious Disease, College of Veterinary Medicine, Seoul National University, Seoul 08826, Korea; irene85537@gmail.com (H.-E.P.); sujeeksj43@snu.ac.kr (S.K.); jin08061992@snu.ac.kr (S.S.); twinstar23@snu.ac.kr (H.-T.P.); daydew@snu.ac.kr (W.B.P.); bini@snu.ac.kr (Y.B.I.); 2 Department of Pathobiology and Diagnostic Investigation, Michigan State University, East Lansing, MI 48824, USA; 3 BK21 PLUS Creative Veterinary Research Center, Seoul National University, Seoul 08826, Korea; 4 Research Institute for Veterinary Science, Seoul National University, Seoul 08826, Korea; 5 Bio-Max/N-Bio Institute, Seoul National University, Seoul 08826, Korea

**Keywords:** antibiotic resistance, aminoglycoside resistance, environmental mycobacteria, macrolide resistance, multidrug resistance, nontuberculous mycobacteria

## Abstract

Non-tuberculous mycobacteria (NTM) are ubiquitous microorganisms that have the potential to cause disease in both humans and animals. Recently, NTM infections have rapidly increased in South Korea, especially in urbanized areas. However, the distribution of species and the antibiotic resistance profile of NTM in environmental sources have not yet been investigated. Therefore, we analyzed the distribution of species and the antibiotic resistance profile of NTM in soil within urban areas of South Korea. A total of 132 isolates of NTM were isolated from soil samples from 1 municipal animal shelter and 4 urban area parks. Among the 132 isolates, 105 isolates were identified as slowly growing mycobacteria (SGM) and 27 isolates as rapidly growing mycobacteria (RGM) based on the sequences of the *rpoB* and *hsp65* genes. The antibiotic resistance patterns of NTM isolates differed from species to species. Additionally, a mutation in the *rrs* gene found in this study was not associated with aminoglycoside resistance. In conclusion, our results showed that NTM isolates from South Korean soil exhibit multidrug resistance to streptomycin, amikacin, azithromycin, ethambutol, isoniazid, and imipenem. These results suggest that NTM may pose a public threat.

## 1. Introduction

Non-tuberculous mycobacteria (NTM) are ubiquitous bacteria that are widely distributed in natural environments such as water, soil, and dust [1,2]. NTM have been considered saprophyte and colonizer microbes [1,2]. However, some NTMs are opportunistic pathogens that have the potential to cause diseases in immune-compromised hosts [3,4,5]. NTM-associated diseases are classified into four distinct clinical types: pulmonary disease, lymphadenitis, cutaneous disease, and disseminated disease [5]. NTM infection mostly occurs by exposure to environmental sources of NTM such as soil, water, and dust [3,4,5]. In contrast, the person-to-person transmission of NTM infection is not common [6]. Generally, after the ingestion of NTM through the respiratory system, NTM are cleared from the host by the immune system, and infection is not established. However, predisposing factors such as repetitive exposure to NTM, an immunosuppressed condition, and the genetic susceptibility of the host lead to chronic infection [6,7,8].

According to previous studies, the adaptation of NTM in human-associated and household environments such as water distribution systems, bathtubs, and showerheads has been reported [2,3,9,10]. Several characteristics of NTM, such as a slow growth rate, lipid-rich outer membrane, hydrophobic cells, and biofilm formation, lead to the persistence of NTM in human-associated and household environments and therefore, increase the possibility of exposure to a host [11,12,13]. For example, NTMs are resistant to chlorine, which is used for water disinfection, through biofilm formation [14]. Repetitive exposure to NTM in environmental sources can threaten public health, especially for immunocompromised populations [15], and the treatment of NTM infection is challenging due to the broad spectrum of resistance to antibiotics in these microbes [16,17]. NTM exhibit a broad range of antibiotic resistance with various mechanisms [16,17]. The mutation of various genes, such as *rpoB*, *katG*, *pncA*, *inhA*, *rrs*, and *rrl*, induces antibiotic resistance in mycobacteria [18,19,20,21,22]. For example, the mutation of the 81-bp region of the *rpoB* gene causes a considerable level of rifampin resistance in both *M. tuberculosis* and NTM [18,21]. Additionally, a point mutation in the *katG* gene interferes with the activation of the pro-drug isoniazid in mycobacteria [20]. Mutations at nucleotide positions 491, 512, 513, 516, 904, and 905 within the *rrs* gene induce changes in the interaction between *rpsL* and 16S rRNA that lead to streptomycin resistance in *M. tuberculosis* [22]. Furthermore, mycolic acid and lipid-rich cell wall components confer considerable antibiotic resistance in NTM through the inactivation of antimicrobial peptides [23].

The prevalence and incidence of human diseases caused by NTM are steadily increasing worldwide [24,25,26,27,28,29,30]. The prevalence of NTM disease and related mycobacterial species differs depending on the country and area. According to the Nontuberculous Mycobacteria Network European Trialsgroup (NTM-NET) collaborative study, the members of the *Mycobacterium avium* complex (MAC) are the predominant mycobacterial species in pulmonary NTM infections in most countries [31]. In the United States, the MAC represents the most frequently isolated NTM species, followed by *M. kansasii* [31]. In South Korea, the number of patients diagnosed with NTM who are treated for lung disease began increasing after the 1980s, and the MAC is predominant in clinical samples from pulmonary NTM infections [32]. *M. abscessus* is the second most dominant species after MAC members in South Korea but has a relatively low prevalence in other countries [28,33].

The emergence of NTM infection worldwide requires an improved understanding of NTM for the establishment of an effective prevention and treatment strategy for NTM disease. Therefore, the ecological investigation of NTM in environmental sources, including the species distribution, genetic diversity, and antibiotic resistance profile, is key to achieving successful control of NTM infection. The species distribution of environmental mycobacteria in South Korea has been investigated in soil, dust, well water, and sewage samples collected from 123 randomly selected areas [34]. However, the antibiotic resistance profile of NTM and its genetic determinants in environmental sources have not been investigated in South Korea. Therefore, we isolated NTM from soil samples collected in 1 animal shelter and 4 urban area parks to investigate the antibiotic resistance profile and its genetic determinants.

## 2. Materials and Methods

### 2.1. Sampling

Soil samples were obtained from 1 animal shelter in Incheon and 4 urban area parks in Seoul, where companion animals can be let free to stay without a leash. Ten soil samples were taken per site. Soil samples of more than 5 g were collected within 5 cm from the surface. The soil samples were transported to the laboratory and immediately processed for the isolation of NTM.

### 2.2. Isolation of NTM from Soil Samples

Five grams of each soil sample was transferred to a 50 mL tube containing 30 mL of PBS, which was then vortexed for 1 min. After standing at room temperature for 30 min, 15 mL of the suspension was added to a new 50 mL tube, which was then centrifuged at 3000× *g* for 10 min. After centrifugation, the pellet was resuspended in 1 mL of PBS. Thereafter, 2 mL of a 1 M NaOH solution was added, and the tube was left to stand for 20 min at room temperature. Thirty mL of distilled water were added for neutralization, followed by centrifugation for 10 min at 3000× *g*. After centrifugation, the pellet was suspended in 2 mL of 5% oxalic acid and incubated at room temperature for 20 min. Thirty mL of distilled water were added for final neutralization, followed by centrifugation at 3000× *g* for 10 min. The pellet was resuspended in 1 mL of PBS to wash out any residual decontamination reagent, followed by centrifugation at 10,000× *g* for 5 min. Finally, the pellet was suspended in 150 µL of PBS and inoculated onto 7H9 agar supplemented with polymyxin B (20 mg/L), amphotericin B (10 mg/L), nalidixic acid (10 mg/L), trimethoprim (10 mg/L) and azlocillin (10 mg/L). The inoculated plates were incubated for 4 to 6 weeks at 37 °C. Rapidly growing mycobacteria (RGM) and slowly growing mycobacteria (SGM) were defined as the mycobacteria that grew on the media within or after 7 days, respectively. Colonies suspected of being NTM were transferred to new 7H9 agar without antibiotics to confirm the pure isolation of the strain. Finally, a single colony was transferred to 7H9 broth, followed by incubation at 37 °C for up to 2 weeks and storage at −80 °C for further analysis.

### 2.3. Extraction of Mycobacterial DNA

DNA extraction was conducted as previously described with slight modification [35]. Briefly, 1 mL of bacterial culture was transferred to a 1.5 mL tube, followed by centrifugation at 10,000× *g* for 5 min. The bacterial pellet was resuspended in 500 µL of guanidine thiocyanate L6 lysis buffer (5.25 M guanidine thiocyanate, 50 mM Tris-HCl (pH 6.4), 20 mM EDTA, 1.3% Triton X-100, distilled water) and incubated at 95 °C for 15 min. The tube was transferred to −20 °C and incubated for 5 min. Thereafter, the mixture was vortexed and centrifuged at 13,000× *g* for 1 min. After centrifugation, 300 µL of supernatant was transferred into a new 1.5 mL tube containing 700 µL of L6 lysis buffer. The mixture was vortexed and incubated at 70 °C for 5 min. After incubation, 250 µL of 100% ethanol was added, followed by incubation at 56 °C for 5 min after vortex. The mixture was transferred to a DNA spin column (Elpis Bio, Daejeon, Korea), which was then centrifuged at 13,000× *g* for 1 min. The column was washed with 700 µL of L2 washing buffer (5.25 M guanidine thiocyanate, 50 mM Tris-HCl, distilled water) and washed twice, again with 700 µL of 70% ethanol. Finally, 100 µL of nuclease-free water was added to the column, followed by centrifugation at 13,000× *g* for 1 min for the elution of DNA. Purified DNA was stored at −20 °C until use.

### 2.4. Sequence-Based Identification of Environmental NTM Isolates

The amplification of the 16S rRNA, *hsp65*, and *rpoB* genes was performed following previous studies [36,37,38]. The PCR mixture for the 16S rRNA, *hsp65*, and *rpoB* genes consisted of 5 µL of 10× i-Taq PCR buffer (Intron, Gyeonggi-do, Korea), 4 µL of 10 mM deoxynucleotide triphosphates (dNTPs), 1 µL of forward and reverse primers at 10 µM, 2.5 U of i-Taq DNA polymerase (Intron, Gyeonggi-do, Korea), 36.5 µL of nuclease-free water and 2 µL of DNA in a total volume of 50 µL. First, PCR for 16S rRNA was conducted as follows: 95 °C for 8 min, 29 cycles of 95 °C for 60 s, 60 °C for 40 s, 72 °C for 35 s and final extension at 72 °C for 10 min. DNA samples that were positive for 16S rRNA were tested for the *hsp65* and *rpoB* genes by PCR. PCR for the *hsp65* gene was conducted as follows: 94 °C for 5 min, 35 cycles of 95 °C for 30 s, 60 °C for 30 s, 72 °C for 60 s and a final extension at 72 °C for 10 min. PCR for the *rpoB* gene was conducted as follows: 95 °C for 5 min, 35 cycles of 94 °C for 30 s, 64 °C for 30 s, 72 °C for 90 s and a final extension at 72 °C for 5 min. All PCR assays were carried out with a Veriti Thermal Cycler (Applied Biosystems, Foster City, CA, USA). Electrophoresis was performed on a 1.5% agarose gel, and the results were visualized with a UV transilluminator. Amplicons were purified with a Big Dye Terminator v3.1 cycle sequencing kit (Applied Biosystems, Foster City, CA, USA) and sequenced using an Applied Biosystems 3730xl DNA Analyzer. The DNA sequences were aligned by using MEGA software version 10.0 (available online: https://www.megasoftware.net/). Sequence analysis of the aligned DNA sequences of *hsp65* and *rpoB* was performed using NCBI BLAST (available online: https://blast.ncbi.nlm.nih.gov). The primers used for the identification of NTM are listed in Table 1.

### 2.5. Phylogenetic Tree Analysis

Sequences of the *hsp65* and *rpoB* genes were trimmed to start and end at the same nucleotide position for all isolates. The alignment of multiple sequences was conducted with MEGA software. The phylogenetic analysis was performed based on 413 bp of *hsp65* and 617 to 626 bp *rpoB* gene sequences by using MEGA software. The phylogenetic tree was constructed from the DNA sequences by using the neighbor-joining method, and the evolutionary distances were computed using the Jukes-Cantor method.

### 2.6. Antibiotic Resistance Test

Antibiotic susceptibility testing against 8 antibiotics (rifampin (RIF), streptomycin (STR), amikacin (AMK), azithromycin (AZI), ethambutol (ETH), isoniazid (INZ), Moxifloxacin (MXF) and Imipenem (IMP)) was performed by the broth microdilution method, as previously described [42,43]. The minimum inhibitory concentration was read at 7 and 14 days for SGM and 3 and 7 days for RGM. Interpretation of the results was performed by following the Clinical and Laboratory Standards Institute (CLSI M24-A2) guidelines. The *Mycobacterium intracellulare* ATCC13950 and *Mycobacterium avium* 104 strains were used as quality controls. The minimum inhibitory concentration (MIC) thresholds of the antimicrobial agents indicating susceptible, intermediate and resistant classifications were interpreted according to the CLSI guidelines (Table 2).

### 2.7. PCR and Sequence Analysis Associated with Antibiotic Resistance

The 16S rRNA (*rrs*) gene and 23S rRNA (*rrl*) gene were selected for correlation analysis with antibiotic resistance and gene mutation. The amplification of the *rrs* and *rrl* genes was performed as previously described [39,40]. Extracted genomic DNA from the NTM isolates was used as a DNA template. The PCR mixture consisted of 5 µL of 10× i-Taq PCR buffer, 4 µL of 10 mM dNTPs, 1 µL of forward and reverse primers at 10 µM, 2.5 U of i-Taq DNA polymerase, 36.5 µL of nuclease-free water and 2 µL of DNA in a total volume of 50 µL. First, PCR for *rrs* was conducted as follows: 94 °C for 10 min, 35 cycles of 94 °C for 30 s, 55 °C for 30 s, 72 °C for 60 s and followed by 5 min at 72 °C for final extension. The amplification of *rrl* gene was performed as follows: 95 °C for 10 min, 35 cycles of 94 °C for 1 min, 55 °C for 1 min and 72 °C for 1 min, followed by 7 min at 72 °C for final extension. The PCR products were purified and sequenced using the Big Dye Terminator v3.1 cycle sequencing kit, and sequencing was performed using an Applied Biosystems 3730xl DNA Analyzer. The alignment of sequenced nucleotides was performed with MEGA software (version 10.0). The amplification of the erythromycin ribosome methylase (*erm*) gene was performed as previously described [41]. PCR was conducted with the following steps: 94 °C for 2 min, followed by 35 cycles at 94 °C for 30 s, 60 °C for 30 s and 72 °C for 30 s and a final extension of 5 min at 72 °C. The primers used in the PCR and sequence analyses associated with antibiotic resistance are listed in Table 1.

## 3. Results

### 3.1. Isolation and Identification of NTM from Soil Samples

**Figure 1:**
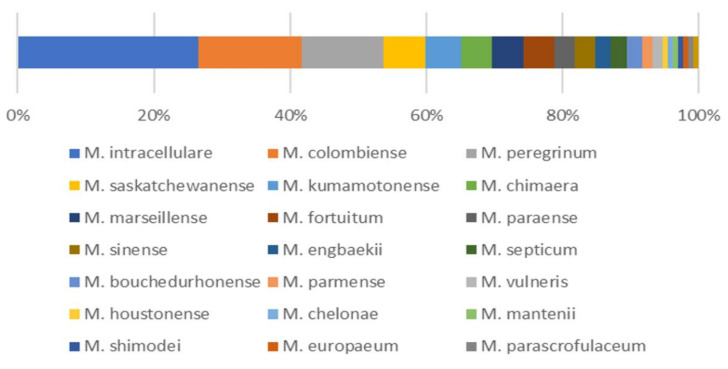
Distribution of non-tuberculous mycobacterial species isolated from South Korean soils.

A total of 132 isolates of NTM were isolated from 50 soil samples from 5 sites, 105 of which were SGM, and the remaining 27 isolates were RGM. Among the 132 isolated isolates, 22 different NTM species were identified (Figure 1). The predominant species was *M. intracellulare* (*n* = 35, 26.5%). The second and third most frequently isolated species were *M. colombiense* (*n* = 20, 15.2%) and *M. peregrinum* (*n* = 16, 12.1%), respectively. Other less frequently isolated species and the identified numbers of isolates were as follows: *M. saskatchewanense* (*n* = 8), *M. kumamotonense* (*n* = 7), *M. chimaera* (*n* = 6), *M. marseillense* (*n* = 6), *M. fortuitum* (*n* = 6), *M. paraense* (*n* = 4), *M. sinense* (*n* = 4), *M. engbaekii* (*n* = 3), *M. septicum* (*n* = 3), *M. bouchedurhonense* (*n* = 3), *M. parmense* (*n* = 2), *M. vulneris* (*n* = 2), *M. houstonense* (*n* = 1), *M. chelonae* (*n* = 1), *M. mantenii* (*n* = 1), *M. shimoidei* (*n* = 1), *M. europaeum* (*n* = 1), *M. parascrofulaceum* (*n* = 1) and *M. genavense* (*n* = 1).

### 3.2. Phylogenetic Tree Analysis

**Figure 2:**
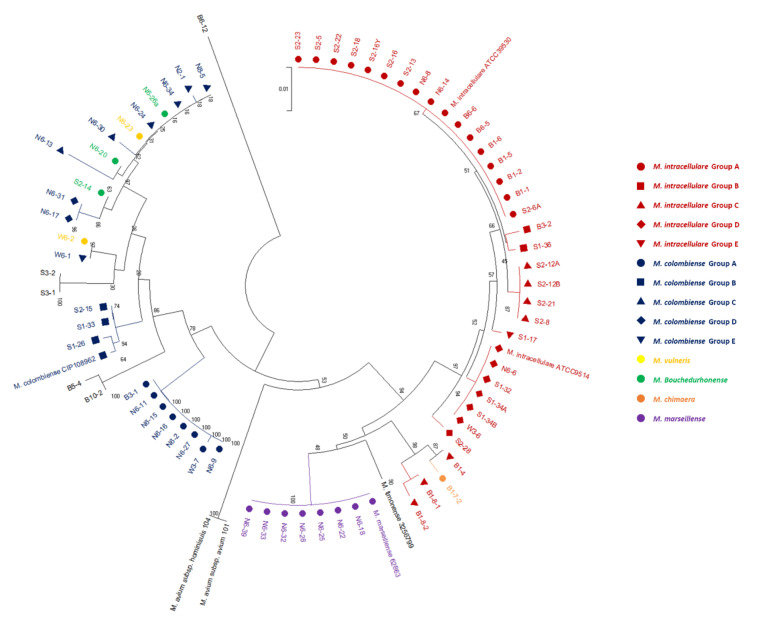
Phylogenetic analysis of *Mycobacterium avium* complex (MAC) isolates in South Korean soils based on the *rpoB* gene sequences of the isolates and previously reported strains in NCBI GenBank. The tree was created using the neighbor-joining method, and bootstrap analysis was performed from 1000 replications.

**Figure 3:**
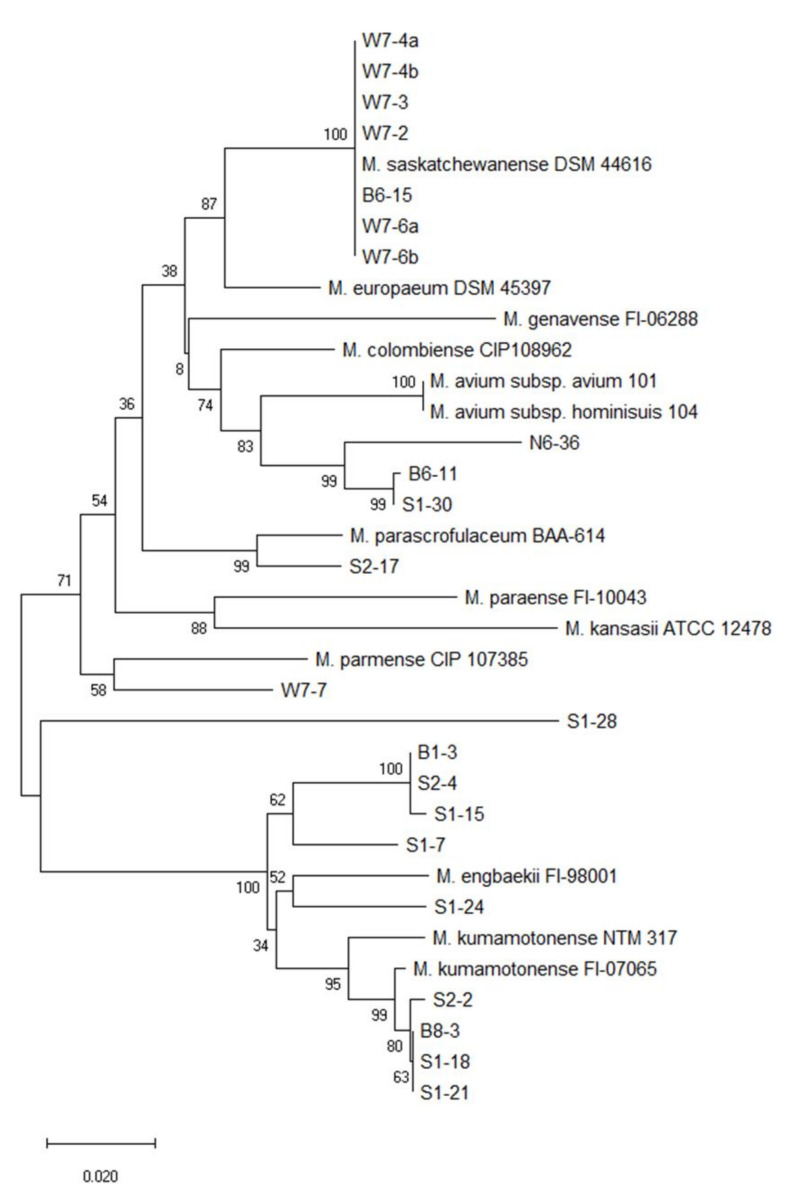
Phylogenetic analysis of non-MAC SGM isolates in South Korean soils based on the *rpoB* gene sequences of the isolates and previously reported strains in NCBI GenBank. The tree was created using the neighbor-joining method, and bootstrap analysis was performed from 1000 replications.

**Figure 4:**
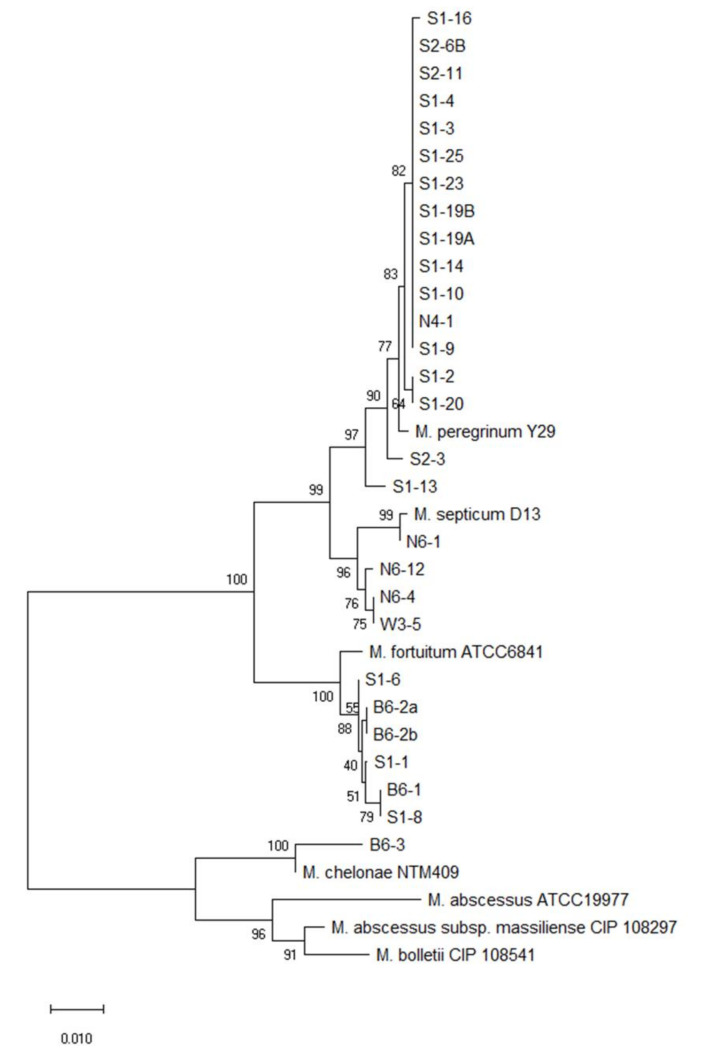
Phylogenetic analysis of RGM isolates in South Korean soils based on the *rpoB* gene sequences of the isolates and previously reported strains in NCBI GenBank. The tree was created using the neighbor-joining method, and bootstrap analysis was performed from 1000 replications.

**Figure 5:**
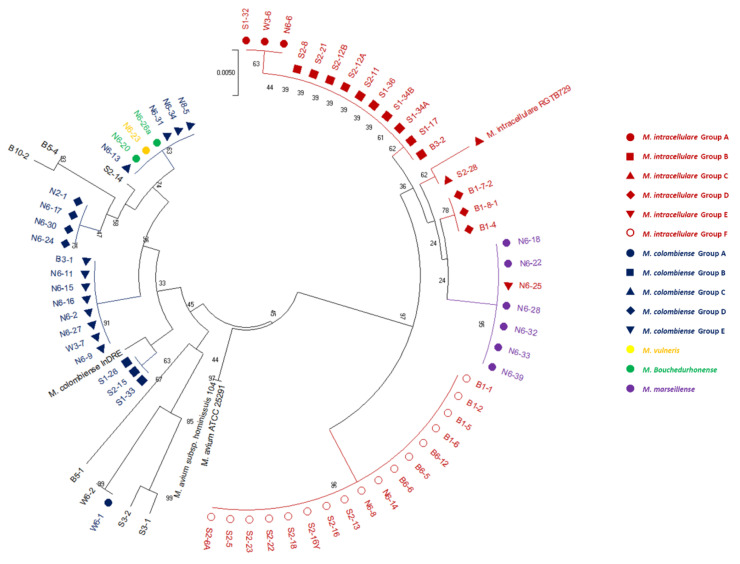
Phylogenetic analysis of MAC isolates in South Korean soils based on the *hsp65* gene sequences of the isolates and previously reported strains in NCBI GenBank. The tree was created using the neighbor-joining method, and bootstrap analysis was performed from 1000 replications.

**Figure 6:**
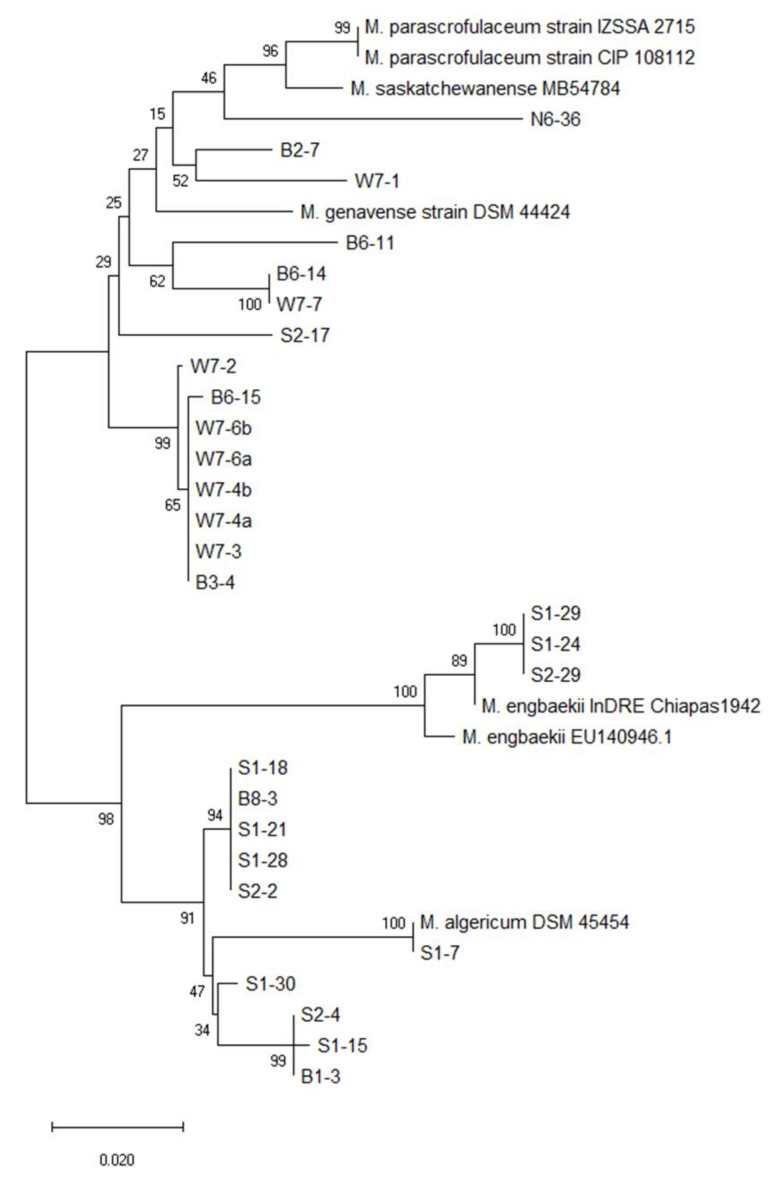
Phylogenetic analysis of non-MAC SGM isolates in South Korean soils based on the *hsp65* gene sequences of the isolates and previously reported strains in NCBI GenBank. The tree was created using the neighbor-joining method, and bootstrap analysis was performed from 1000 replications.

**Figure 7:**
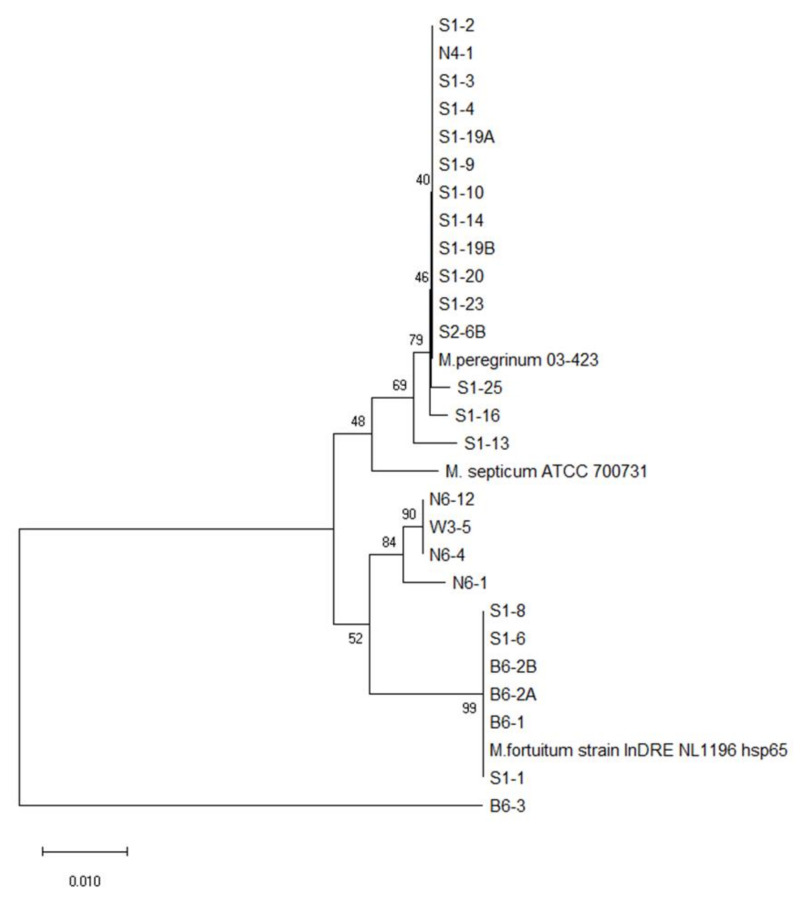
Phylogenetic analysis of RGM isolates in South Korean soils based on the *hsp65* gene sequences of the isolates and previously reported strains in NCBI GenBank. The tree was created using the neighbor-joining method, and bootstrap analysis was performed from 1000 replications.

Phylogenetic analysis based on the *rpoB* and *hsp65* genes revealed very close genetic similarity in NTM isolates (Figure 2, Figure 3, Figure 4, Figure 5, Figure 6 and Figure 7). In the analysis of the *rpoB* gene of MAC isolates, 32 *M. intracellulare* isolates were divided into five groups (Figure 2). Additionally, three isolates (B1-4, B1-8-1 and B1-8-2) showed high similarity to one *M. chimaera* isolate (B1-7-2). Six isolates of *M. marseillense* were identical to the previously reported *M. marseillense* 62863 strain. Twenty isolates of *M. colombiense* were classified into five groups, and one group was closely related to the *M. colombiense* CIP108962 strain (Figure 2). Three other groups were related to *M. vulneris* and *M. bouchedurhonense* isolates. In the phylogenetic tree analysis with non-MAC SGM species, most isolates were closely related to previously reported isolates (Figure 3). Additionally, four isolates of *M. kumamotonense* (S2-2, B8-3, S1-18 and S1-21) were closely related to the previously reported *M. kumamotonense* FI-07065 strain. Seven isolates of *M. saskatchewanense* were closely related to the *M. saskatchewanense* DSM 44616 strain. Among RGM species, most of the *M. peregrinum* isolates belonged to one cluster and were related to the *M. peregrinum* Y29 strain based on the *rpoB* gene sequence. Additionally, six isolates of *M. fortuitum* were classified into one cluster that was closely related to *M. fortuitum* ATCC6841 strain (Figure 4). The analysis of the *hsp65* gene revealed similar results to the analysis of the *rpoB* gene (Figure 5). The *M. intracellulare* isolates were divided into six groups, and one isolate (N6-25) belonged to a single cluster with six *M. marseillense* isolates. Twenty isolates of *M. colombiense* were divided into four clusters, and one isolate (W6-1) was closely related to *M. vulneris* isolates. In the phylogenetic tree based on the *hsp65* gene sequence of non-MAC SGM isolates (Figure 6), seven isolates of *M. saskatchewanense* clustered into one group that was not closely related to *M. saskatchewanense* MB54784 strain, whereas a close relationship was indicated in the analysis of the *rpoB* gene. Additionally, three *M. engbaekii* isolates were closely related to the previously reported *M. engbaekii* InDRE Chiapas 1942 strain. As inferred from the *hsp65* gene sequences of the RGM isolates, 15 isolates of *M. peregrinum* were classified into two clusters, and one cluster was closely related to the *M. peregrinum* 03-423 strain. Furthermore, six isolates of *M. fortuitum* were classified into a single cluster that was closely related to the *M. fortuitum* InDRE NL1196 strain. Three isolates of *M. septicum* classified into a single group that was not closely related to the previously reported *M. septicum* ATCC 700731 strain.

### 3.3. Antibiotic Resistance Tests

Among the 132 isolates, 118 isolates were resistant to at least one antibiotic, and 14 isolates were susceptible to all tested antibiotics. Among the total 132 isolates, 107 isolates showed antibiotic resistance to INZ (81%). On the other hand, only 5 out of the 132 isolates showed antibiotic resistance to MXF (3.7%). Other antibiotics showed the following resistance rates: STR (45.8%), AMK (23.3%), RIF (12.7%), AZI (27.1%), ETH (24.1%) and IMP (56.4%). Among the 118 isolates showing antibiotic resistance, 63 isolates showed multidrug resistance, indicating resistance to three or more antibiotic classes.

Antibiotic resistance was significantly different depending on the mycobacterial species (Supplementary Table S1). Among the 35 *M. intracellulare* isolates, 34 isolates were resistant to at least one antibiotic, and 82.8% of the isolates were multidrug-resistant. In addition, all isolates of *M. kumamotonense* and *M. engbaekii* were multidrug-resistant. In contrast, 8 out of 20 isolates of *M. colombiense* were susceptible to all antibiotics, and only 15% of all isolates showed multidrug resistance. In addition, no multidrug-resistant isolates were detected in three mycobacterial species (*M. marseillense*, *M. bouchedurhonense* and *M. vulneris*). A similar pattern was found in the RGM. All isolates of *M. fortuitum* were resistant to azithromycin and showed multidrug resistance. On the other hand, only one isolate of *M. peregrinum* was resistant to azithromycin, and 43.8% of the *M. peregrinum* isolates were multidrug-resistant. Both SGM and RGM exhibited high resistance rates to isoniazid, while the SGM presented higher resistance rates against imipenem than the RGM (68.9% to 18.5%). The following MIC90 values were observed: SGM (RIF: 2 μg/mL, STR: 64 μg/mL, AMK: 128 μg/mL, AZI: 64 μg/mL, ETH: 16 μg/mL, INZ: 128 μg/mL, MXF: 2 μg/mL, IMP: 256 μg/mL) and RGM (RIF: 16 μg/mL, STR: 32 μg/mL, AMK: 16 μg/mL, AZI: 256 μg/mL, ETH: 512 μg/mL, INZ: 256 μg/mL, MXF: 0.25 μg/mL, IMP: 256 μg/mL). The distribution of MIC values varied depending on the mycobacterial species ( Supplementary Tables S2 and S3). However, SGM tended to be more resistant to streptomycin, amikacin and imipenem, whereas RGM tended to be more susceptible.

### 3.4. PCR and Sequence Analysis Associated with Antibiotic Resistance

The mutation of *rrs* and *rrl* genes was investigated by sequencing parts of the two genes that are related to antibiotic resistance. Six mutation types were found in six isolates, and one isolate harbored two mutation types (Table 3). Two mutation types were detected at positions 1190 and 1446 of the *rrs* gene in three *M. intracellulare* isolates. Additionally, four mutation types were found at positions 1191, 1235, 1513 and 1520 in three NTM species (*M. colombiense*, *M. peregrinum*, and *M. sinense*). On the other hand, only one type of mutation within the *rrl* gene was found at position 2419 in six isolates of *M. intracellulare*. With the exception of one isolate, all other isolates were resistant to azithromycin (Table 3). The *erm* gene, which is related to macrolide resistance, was detected in six isolates of *M. fortuitum* and one isolate of *M. houstonense*.

## 4. Discussion

Pulmonary infections caused by various NTM species have affected massive populations and are continuously increasing worldwide [24,25,26,27,28,29,30]. In the United States, the prevalence of NTM lung disease increased significantly from 20 to 47 cases per 100,000 persons from 1997 to 2007 [44]. Additionally, NTM-related death rates not associated with HIV infection significantly increased, while tuberculosis death rates continuously decreased in the United States from 1999 to 2014 [45]. A similar burden of NTM disease has been found in other nations [46,47,48]. In South Korea, the prevalence of tuberculosis fell from 106.5 to 74.4 cases, while the prevalence of NTM infection increased from 9.4 to 36.1 cases per 100,000 population from 2009 to 2016 [46]. Similarly, the number of deaths related to NTM infection increased from 3 to 1121 from 1970 to 2010 in Japan [47]. The NTM-related mortality rate increased from 0.003 to 0.128 per 100,000 population during the same period [47]. In Germany, the prevalence of NTM pulmonary disease increased from 2.3 to 3.3 cases per 100,000 population between 2009 and 2014 [48]. Taken together, the available evidence indicates that the emergence of NTM infection is a global trend that represents a risk to public health.

The MAC members are the most frequently isolated NTM species worldwide, and *M. avium* accounts for the largest portion of clinical isolates, followed by other MAC members such as *M. chimaera*, *M. intracellulare*, *M. marseillense* and *M. colombiense* [49,50]. In our study, the most frequently isolated NTM species was *M. intracellulare*, followed by *M. colombiense*, *M. chimaera* and *M. marseillense*. In contrast, *M. avium* subsp. *avium* was not isolated in the current study. Additionally, *M. kansasii* and *M. abscessus*, which are frequently isolated from clinical samples, were not isolated. The distribution of NTM species can be affected by environmental factors such as nutrients, acidity and the aridity of soil [51]. In this context, the absence of several clinically relevant NTM species in the current study might be related to the characteristics of the soil samples and sampling sites.

Antibiotic resistance is a major emerging global issue that can threaten public health and food security [52,53]. Antibiotic resistance of NTM has been described in previous studies [54,55,56,57]. Several studies reported evidence of the transmission of NTM infection from environmental sources [51,58]. Therefore, the antibiotic resistance profile of NTM in environmental sources is key to establishing a treatment and control strategy for NTM infection. Multidrug resistance of NTMs against eight antibiotics was identified in this study.

In the present study, the antibiotic resistance pattern differed depending on the NTM species. Among SGM, the resistance rates for eight antibiotics were higher in *M. intracellulare* than in other MAC members, such as *M. colombiense*, *M. marseillense*, *M. chimaera*, *M. bouchedurhonense* and *M. vulneris*. All *M. engbaekii* isolates were resistant to AMK, STR, INZ, MXF and IMP, while six isolates of *M. saskatchewanense* were only resistant to INZ. Among RGM, the resistance rates to RIF and AZI were higher in *M. fortuitum* than in *M. peregrinum*. Specific cell wall components of NTM may be responsible for these species-specific antibiotic resistance patterns. Mycobacterial glycopeptidolipids (GPLs) are highly antigenic and species- or serovar-specific glycopeptides produced by various NTM species [59,60,61]. The GPLs of NTM share identical lipopeptide cores with different post-translational modifications, such as glycosylation, methylation and acetylation [61]. Collectively, these phenomena might be due to the differences in the permeability of the cell wall of NTM species conferred by the different post-translation mechanisms of glycosylation, methylation and acetylation.

Streptomycin and amikacin are aminoglycoside antibiotics that are commonly used for the treatment of NTM infection [16]. Most reference strains of NTM, including *M. intracellulare* ATCC13950, *M. kansasii* ATCC12478, *M. fortuitum* ATCC6841 and *M. peregrinum* ATCC14467, are susceptible to amikacin [62]. Additionally, a considerable proportion of clinical MAC isolates are susceptible to amikacin and streptomycin [63,64]. However, amikacin- and streptomycin-resistant NTM isolates were isolated in this study. Sixty percent of the *M. intracellulare* isolates, two isolates of *M. kumamotonense* and three isolates of *M. engbaekii* were resistant to both amikacin and streptomycin in our study. Resistance to aminoglycoside antibiotics is associated with mutation of the *rrs* gene at specific sites [22]. The mutation of the *rrs* gene at sites including T1406A, A1408G, C1409T and G1491T confers considerable resistance to amikacin in NTM species [39]. However, no evidence of mutations in the *rrs* gene related to aminoglycoside resistance was found in our NTM isolates. It is possible that other antibiotic resistance mechanisms, such as drug-modifying enzyme-induced resistance to amikacin [56] or mutation of the *rpsL* gene inducing a high level of streptomycin resistance [22], could be involved in the resistance observed in our NTM isolates.

Macrolide antibiotics such as erythromycin, clarithromycin and azithromycin are widely used for the treatment of NTM lung disease [16]. Resistance to macrolide antibiotics in NTM is mainly associated with two mechanisms, involving erythromycin ribosomal methylase (*erm*) [41] and point mutation of the peptidyltransferase domain of the 23S rRNA (*rrl*) gene at specific sites [65]. Among our 36 azithromycin-resistant isolates, the T2419C mutation was identified in five isolates of *M. intracellulare*, and the *erm* gene was detected in six isolates of *M. fortuitum* and one isolate of *M. houstonense*. The rest of these isolates did not harbor any mutations of *rrl* or *erm* genes. Other resistance mechanisms, such as mechanisms involving macrolide esterase [66] and macrolide phosphotransferase [67], have been reported. Additionally, plasmid-mediated macrolide resistance has been identified in clinical and environmental isolates of bacteria [68]. Therefore, the possible involvement of these mechanisms should be investigated in further studies to identify novel macrolide resistance mechanisms in the rest of our NTM isolates.

Isoniazid, ethambutol and rifampicin are first-line anti-tuberculosis drugs that are used for the treatment of mycobacterial infection [69]. In the current study, 81% of the identified NTM were resistant to isoniazid. Our findings are consistent with previous studies indicating a high resistance rate to first-line anti-tuberculosis drugs in the NTM [70,71]. However, the resistance rates of ethambutol and rifampicin were relatively lower than that of isoniazid, which disagrees with a previous study [72].

Carbapenem resistance is an emerging threat to public health worldwide and mainly occurs in pathogens such as *Acinetobacter baumannii*, *Pseudomonas aeruginosa*, *Stenotrophomonas maltophilia*, *Escherichia coli* and *Klebsiella pneumoniae* [73]. The same resistance gene cassettes associated with various antibiotics, such as aminoglycosides, amphenicols, carbapenem, sulfonamides and tetracyclines, are found in both soil bacteria and pathogenic bacteria [74]. Although genetic analysis related to imipenem resistance was not carried out in our NTM isolates, genetic mobile elements related to the enzymatic inactivation of antibiotics, efflux pumps and outer-membrane permeability might be involved.

Moxifloxacin has been widely used for the treatment of NTM lung disease, especially in macrolide-resistant NTM infections [75]. The incidence of moxifloxacin resistance in clinical isolates of NTM has been reported in previous studies [76,77]. Five isolates of moxifloxacin-resistant NTM were isolated in this study: one isolate of *M. intracellulare*, three isolates of *M. engbaekii* and one isolate of *M. septicum*. In contrast to previous studies, the resistance rate of NTM isolates to moxifloxacin in this study was low, indicating that acquisition of moxifloxacin resistance may occur during treatment in hospitals.

## 5. Conclusions

Although limited in the number of sampling sites, our results suggest the extremely broad spectrum of antibiotic resistance in NTM isolates from the soils of urban areas in South Korea. We currently have insufficient knowledge of environmental NTM regarding their species distributions, antibiotic resistance profiles and antibiotic resistance mechanisms. However, our study demonstrates that antibiotic resistance to aminoglycosides and macrolides in NTM isolates largely depends on intrinsic mechanisms, without any genetic changes in the 16S rRNA and 23S rRNA genes. Additionally, NTM isolates that are resistant to isoniazid, ethambutol, rifampicin, moxifloxacin and imipenem were identified. Although we investigated the genetic background only in association with aminoglycoside and macrolide resistance, it can be inferred that antibiotic resistance is related to genetic mobile elements, which might be acquired from other bacteria found naturally in soil. Novel mechanisms and associated factors of antibiotic resistance in environmental NTM isolates should be investigated in further studies to prevent the dissemination of NTM infection and the associated threat to the public.

## Figures and Tables

**Table 1:** Nucleotide sequences of the primers used in this study.

Target Gene	Primer Sequence		Product Size (bp)	Reference
Identification of non-tuberculosis mycobacteria				
16s rRNA	F	ATAAGCCTGGGAAACTGGGT	484	[37]
	R	CACGCTCACAGTTAAGCCGT		
*hsp65*	F	ACCAACGATGGTGTGTCCAT	439	[36]
	R	CTTGTCGAACCGCATACCCT		
*rpoB*	F	GGCAAGGTCACCCCGAAGGG	723	[38]
	R	AGCGGCTGCTGGGTGATCATC		
Identification of antibiotic resistance genes				
*rrs*	F	ATGACGTCAAGTCATCATGCC	341	[39]
	R	AGGTGATCCAGCCGCACCTTC		
*rrl*	F	TTTAAGCCCCAGTAAACGGC	420	[40]
	R	GTCCAGGTTGAGGGAACCTT		
*erm*	F	ACGTGGTGGTGGGCAAYCTG	175	[41]
	R	AATTCGAACCACGGCCACCACT		

**Table 2:** MIC (μg/mL) thresholds of 8 antimicrobial agents for slowly growing mycobacteria (SGM) and rapidly growing mycobacteria (RGM).

Antibiotics	MIC Breakpoints					
	Susceptible		Intermediate		Resistant	
	SGM	RGM	SGM	RGM	SGM	RGM
Rifampicin	≤0.5	<1	1–4	N/A	≥8	≥1
Streptomycin	<5	<5	N/A	N/A	≥5	≥5
Amikacin	≤16	≤16	32	32	≥64	≥64
Azithromycin	≤8	≤2	16	4	≥32	≥8
Ethambutol	≤2	<5	4	N/A	≥8	≥5
Isoniazid	≤0.5	<1	N/A	N/A	≥1	≥1
Moxifloxacin	≤1	≤1	2	2	≥4	≥4
Imipenem	≤4	≤4	8–16	8–16	≥32	≥32

N/A: not applicable.

**Table 3:** Mutations in the *rrs* and *rrl* genes identified by sequencing.

Species	Strain No.	Presence of *erm* Gene	Sequencing Results		MIC Value (μg/mL)		
			*rrs*	*rrl*	STR	AMK	AZI
*M.intracellulare*	S2-16Y	ND	G1190A	WT	0.5	1	0.25
*M.intracellulare*	B1-8-1	ND	G1446T	WT	8	64	32
*M.intracellulare*	B1-4	ND	G1446T	WT	2	16	1
*M.colombiense*	S1-33	ND	C1520G G1513A	WT	0.25	2	1
*M.peregrinum*	S1-3	ND	C1235T	WT	8	2	0.5
*M.sinense*	S2-4	ND	T1191G	WT	128	2	4
*M.intracellulare*	B1-1	ND	WT	T2419C	8	128	32
*M.intracellulare*	B1-6	ND	WT	T2419C	16	128	32
*M.intracellulare*	S2-16	ND	WT	T2419C	8	128	64
*M.intracellulare*	S2-18	ND	WT	T2419C	4	64	16
*M.intracellulare*	S2-22	ND	WT	T2419C	16	128	64
*M.intracellulare*	S2-23	ND	WT	T2419C	4	64	32

STR: streptomycin, AMK: amikacin, AZI: azithromycin, ND: not detected.

## Supplementary Materials

The following are available online at https://www.mdpi.com/2076-2607/8/8/1114/s1, Table S1: Antibiotic resistance patterns of nontuberculous mycobacterial isolate in South Korean soils. Table S2. Minimal inhibitory concentrations of the slowly growing mycobacteria isolates. Table S3. Minimal inhibitory concentrations of the rapidly growing mycobacteria isolates.
