# Supplementary material for: 16S and 23S rRNA Gene Mutation Independent Multidrug Resistance of Non-Tuberculous Mycobacteria Isolated from South Korean Soil
Source: Microorganisms. 2020 Jul 24;8(8):1114. doi: 10.3390/microorganisms8081114 (PMC7465728; doi:10.3390/microorganisms8081114)
Supplement: Supplementary file 1 [file microorganisms-08-01114-s001.pdf]

Table S1. Antibiotic resistance patterns of nontuberculous mycobacterial isolate in South Korean soils

NTM species		Resistant pattern	No. of isolates
MAC	*M.intracellulare*	RIF+STR+AMK+AZI+ETH+INZ+IMP	1
		STR+AMK+AZI+ETH+INZ+MXF+IMP	1
		RIF+STR+AMK+AZI+INZ+IMP	1
		STR+AMK+AZI+ETH+INZ+IMP	5
		STR+AMK+ETH+INZ+IMP	2
		STR+AMK+AZI+INZ+IMP	11
		STR+ETH+INZ+IMP	1
		STR+AZI+INZ+IMP	1
		AMK+AZI+INZ+IMP	1
		AMK+ETH+INZ+IMP	2
		STR+INZ+IMP	2
		ETH+INZ+IMP	1
		INZ+IMP	1
		AMK+IMP	1
		STR+IMP	1
		INZ	1
		IMP	1
		Susceptible to all tested drugs	1
	M.colombiense	STR+INZ+IMP	3
		INZ+IMP	2
		STR+IMP	3
		INZ	3
		IMP	1
		Susceptible to all tested drugs	8
	M.chimaera	ETH+INZ+IMP	1
		INZ+IMP	2
		AZI+IMP	1
		INZ	1
		Susceptible to all tested drugs	1
	M.marseillense	INZ+IMP	5
		Susceptible to all tested drugs	1
	M.bouchedurhonense	STR+IMP	1
		INZ	1
		Susceptible to all tested drugs	1
	M.vulneris	INZ+IMP	1
		Susceptible to all tested drugs	1
	M.kumamotonense	STR+AMK+INZ+IMP	2
		STR+AZI+ETH+INZ+IMP	1
		STR+AZI+INZ+IMP	1

Non-MAC SGM		STR+INZ+IMP	1
		STR+ETH+INZ+IMP	1
		ETH+INZ+IMP	1
	M.saskatchewanense	STR+INZ	1
		INZ	6
		Susceptible to all tested drugs	1
	M.paraense	RIF+STR+AZI+ETH+IMP	1
		STR+INZ	1
		ETH+INZ	1
		INZ	1
	M.engbaekii	STR+AMK+ETH+INZ+MXF+IMP	1
		STR+AMK+INZ+MXF+IMP	1
		STR+AMK+AZI+INZ+MXF+IMP	1
	M.parmense	INZ+IMP	1
		INZ	1
	M.sinense	INZ+IMP	2
		STR+INZ+IMP	1
		INZ	1
	M.genavense	INZ	1
	M.parascrofulaceum	INZ+IMP	1
	M.europaeum	IMP	1
	M.shimodei	INZ+IMP	1
	M.mantenii	STR+INZ+IMP	1
RGM	*M.fortuitum*	RIF+STR+AZI+INZ	1
		RIF+AZI+INZ	1
		RIF+STR+AZI+ETH+INZ	1
		RIF+STR+AZI+INZ	2
		RIF+AZI+INZ	1
	M.peregrinum	RFI+STR+ETH+INZ	2
		STR+AZI+ETH+INZ	1
		RIF+ETH+INZ	1
		RIF+STR+INZ	2
		RIF+INZ+IMP	1
		ETH+INZ	4
		STR+INZ	1
		INZ+IMP	1
		INZ	2
		STR	1
	M.septicum	RIF+STR+ETH+INZ+MXF+IMP	1
		AZI+INZ+IMP	1
		ETH+INZ	1
	M.houstonense	STR+ETH+INZ+MXF+IMP	1

	M.chelonae	RIF+STR+AZI+ETH+INZ+IMP	1

Table S2. Minimal inhibitory concentrations of the slowly growing mycobacteria isolates

Range of MIC (g/ml)	0.0625	0.125	0.25	0.5	1	2	4	8	16	32	64	128	256	512	>512
RIF	37	7	24	10	13	8	4	1	-	1	1	-			
STR			24	5	13	7	9	14	11	2	12	5	2	1	1
AMK			11	4	18	19	3	6	5	10	17	10	-	3	-
AZI			8	9	16	15	16	6	9	16	7	1	1	2	-
ETH			9	8	21	33	15	6	5	5	1	-	-	1	2
INZ			13	9	9	15	14	10	12	11	1	3	7	1	1
MXF	29	10	29	9	15	10	-	-	1	-	1	2			
IMP			8	3	2	5	2	4	9	8	13	11	38	3	

Table S3. Minimal inhibitory concentrations of the rapidly growing mycobacteria isolates

Range of MIC (g/ml)	0.0625	0.125	0.25	0.5	1	2	4	8	16	32	64	128	256	512	>512
RIF	2	1	3	7	3	4	2	1	3	1					
STR			-	1	-	6	6	7	4	2	1	-	-	-	
AMK			3	-	3	12	2	3	3	1	-	-	-	-	
AZI			4	7	4	1	1	2	1	-	1	1	5	-	
ETH			1	-	1	7	6	3	5	-	-	-	-	1	3
INZ			-	-	3	1	6	2	1	1	6	2	3	-	2
MXF	13	-	12	1	-	-	-	-	-	1	-	-			
IMP			-	1	4	3	5	3	6	1	-	-	4		
